# Effect of Dialysis on the Osmotic Pressure, Conductivity, and Rheology of Aqueous Polyelectrolyte Solutions

**DOI:** 10.1021/acsapm.6c00957

**Published:** 2026-06-15

**Authors:** Bahar Baniasadi, Arva Tejas Desai, Zitan Huang, Victoria Devine-Ducharme, Carlos G. Lopez, Ralph H. Colby

**Affiliations:** † Department of Chemical Engineering, 8082 The Pennsylvania State University, University Park, Pennsylvania 16802, United States; ‡ Department of Mechanical Engineering, The Pennsylvania State University, University Park, Pennsylvania 16802, United States; § Department of Materials Science and Engineering, The Pennsylvania State University, University Park, Pennsylvania 16802, United States; ∥ Department of Biomedical Engineering, The Pennsylvania State University, University Park, Pennsylvania 16802, United States

**Keywords:** polycation, residual salts, viscosity, fraction of free counterions, osmotic coefficient

## Abstract

Dialysis, as a purification step, removes residual salts present in as-received commercial polyelectrolytes. Poly­(diallyldimethylammonium chloride) (PDADMAC) samples, for example, contain substantial amounts of salts from the manufacturing process. PDADMAC is a water-soluble polycation with high charge density, a stable quaternary ammonium structure, and broad pH tolerance. The presence of residual salts increases the ionic strength and can significantly influence measured solution properties. In this work, we compare the viscosity, osmotic pressure measured by freezing point depression, and conductivity of aqueous solutions of dialyzed PDADMAC and its copolymer with acrylamide, poly­(acrylamide-*co*-diallyldimethylammonium chloride), with those of their as-received, not dialyzed counterparts to assess the influence of residual salts and impurities on these polyelectrolytes. The residual salts dominate the measured osmolality, so osmotic pressure cannot be used to determine the fraction of dissociated counterions for samples that have not been properly dialyzed to remove residual salts. The presence of residual salts also significantly lowers the solution viscosity of the as-received samples compared with that of the dialyzed samples at the same polymer concentration. The dialyzed samples do not show the expected concentration dependences of correlation length, suggesting that PDADMAC and its copolymers are likely branched polyelectrolytes.

## Graphical abstract

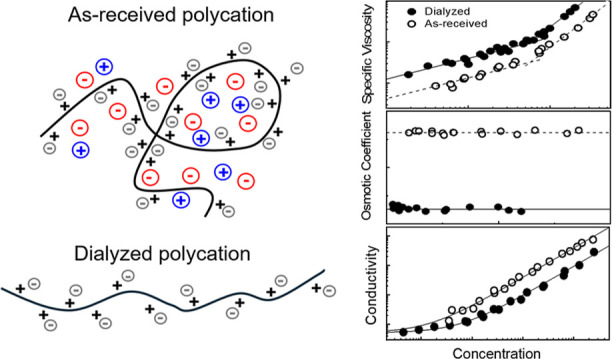

## 1 Introduction

The presence of salts in polyelectrolyte solutions leads to electrostatic screening, which directly alters chain conformation and solution viscosity.^1–5^ Commercial polyelectrolytes often carry considerable residual salt content. For instance, sodium polystyrenesulfonate (NaPSS) can contain substantial amounts of inorganic sulfate and sodium chloride impurities depending on the sulfonation medium.^6,7^ Residual salts in NaPSS solutions significantly lower the specific viscosity,^8,9^ and increase surface tension and osmotic coefficient^6^ by contributing additional osmotically active ions. This leads to an overestimation of the fraction of monomers bearing an effective charge, *f*.^9^

Various studies reporting rheology, scattering, conductivity, and dielectric relaxation of polyelectrolyte solutions describe measurements performed on as-received materials without any purification prior to characterization.^10–18^ The presence of ionic impurities in flexible polyelectrolyte solutions lowers the Debye screening length, the coil size, intermolecular interactions, and many dynamic properties of the chain such as specific viscosity and terminal relaxation time.^1,5^ As a result, the reported physical parameters obtained from measurements of polyelectrolyte solutions reflect not only the intrinsic features of the polyelectrolyte itself, but also uncontrolled variations in salt content. Therefore, purification of commercial polyelectrolytes prior to characterization is critical to eliminate uncontrolled ionic impurities that can bias measured properties. However, for sufficiently rigid polyelectrolytes such as xanthan, this bias can be negligible because when the Kuhn length, *b*, exceeds the Debye screening length, *r*_D_, the chain dimensions become independent of salt concentration.^19–21^

The present work examines how purification affects the viscosity, osmotic coefficient, and conductivity of aqueous solutions of poly­(diallyldimethylammonium chloride) (PDADMAC) and its copolymer with acrylamide, poly­(acrylamide-*co*-diallyldimethylammonium chloride) (PAAcDMAC).

## 2 Background Theory

In semidilute polyelectrolyte solutions with monovalent salt, the salt dependence of properties can be expressed in terms of ${\left(1+\frac{2c_{s}}{fc_{p}}\right)}^{\alpha }$ relating measured quantities to their salt-free values.^1^ In this expression, *f* is the fraction of monomers with dissociated counterions, *c*_p_ is the monomer (repeat unit) number density, *c*_s_ is the number density of monovalent salt molecules, and α is the scaling exponent. The term 2*c*_s_/*fc*_p_ is the ratio of number densities of salt ions and dissociated counterions, with 2*c*_s_/*fc*_p_ ≪ 1 and 2*c*_s_/*fc*_p_ ≫ 1 the low and high salt limits, respectively. Semidilute solutions of linear polyelectrolytes, ( ${c_{p}>c_{p}}^{*}$ ), exhibit a correlation length, ξ, which in a solution with added monovalent salt is given by^1,5,22,23^

1 $$\xi \approx b{\left({c_{p}b}^{3}\right)}^{-1/2}B^{1/2}{\left(1+\frac{2c_{s}}{fc_{p}}\right)}^{1/4}\;\text{for}\;{\;c}_{p}>{c_{p}}^{*}$$

where *b* is the monomer (repeat unit) length, *B* is the dimensionless chain contraction factor, and *c*_p_* is the overlap concentration.

In the semidilute unentangled regime without added salt, the specific viscosity, η_sp_, scales as $\eta_{sp}∼N{\left(c_{p}b^{3}\right)}^{1/2}$ , where *N* is the number of monomers in the chain.^5^ When salt is present, the salt dependence for the viscosity is predicted to scale as^1,5,22,23^

2 $$\eta_{sp}\approx N{\left(c_{p}b^{3}\right)}^{1/2}{B^{-3/2}\left(1+\frac{2c_{s}}{fc_{p}}\right)}^{-3/4}\;\text{for}\;{{{\;c}_{p}}^{*}<c}_{p}<c_{e}$$

assuming the Rouse model applies, where *c*_e_ is the entanglement concentration. Normalizing η_sp_ by $N{\left(c_{p}b^{3}\right)}^{1/2}$ for NaPSS aqueous solutions in Figure 1 shows a viscosity crossover near $c_{p}\approx \frac{2c_{s}}{f}$ , where the solution transitions between the low and high salt limits.

**Figure 1:**
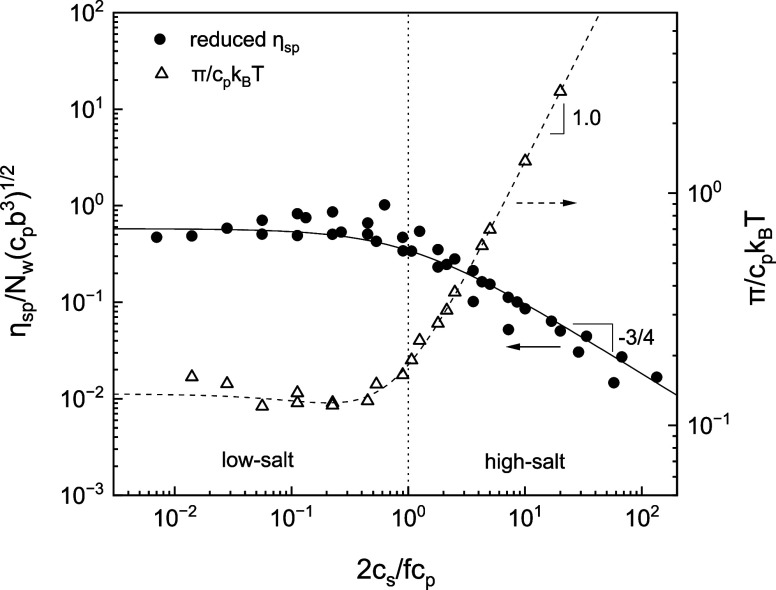
Reduced specific viscosity (filled circles, left axis) and dimensionless osmotic coefficient (open triangles, right axis) for aqueous dialyzed NaPSS (*M*_w_ = 1.2 × 10^6^ g/mol) plotted versus 2*c*_s_/*fc*_p_. Here *c*_s_ is the monovalent salt concentration, and *N*_w_ is the weight-average number of monomers per chain. The value of *f* = 0.137 is obtained by fitting osmotic pressure data to eq 3 (dashed line, right axis). The value of *B* = 1.44 is obtained by fitting specific viscosity data to eq 2 (solid line, left axis). The vertical dotted line indicates the crossover between the low-salt and high-salt regimes. Data adapted from ref 24.

In the concentration range where the polymeric contribution to the osmotic pressure, *k*_B_ *T*/ξ^3^, is negligible compared with the mobile counterion and salt ion contributions, the osmotic pressure, π, of a polyelectrolyte solution can be written as^5^

3 $$\frac{\pi }{k_{B}T}=\frac{{\left({fc}_{p}\right)}^{2}}{4c_{s}+fc_{p}}+2c_{s}$$

where *k*_B_ is the Boltzmann constant, and *T* is the absolute temperature.^1,5^

When π is defined in terms of the total ideal gas pressure of mobile ions in the polyelectrolyte solution in Donnan equilibrium with an external salt reservoir across a membrane, the Donnan expression can be used as follows^1^

4 $$\frac{\pi }{k_{B}T}=2c_{s}\sqrt{{\left(\frac{fc_{p}}{2c_{s}}\right)}^{2}+1}$$

which has a slightly broader crossover between the same two high and low salt limits compared to eq 3.

Figure 1 shows the dimensionless osmotic coefficient, $\varphi =\frac{\pi }{c_{p}k_{B}T}$ , versus $\frac{2c_{s}}{fc_{p}}$ for aqueous NaPSS solutions, and also shows the crossover from the counterion regime to the added salt regime.

## 3 Materials and Methods

### 3.1 Chemicals

Table 1 lists the PDADMAC and PAAcDMAC samples used in this work, including supplier and trade name information. The PDADMAC from Lubrizol is sold under the trade name Merquat 100. The PAAcDMAC copolymers, including Merquat 2200, Merquat 550, and Merquat 740, were also provided by Lubrizol. The mole fraction of DADMAC in the PAAcDMAC copolymers was determined by ^1^H NMR spectroscopy. Details of the molecular weight reported by the vendors can be found in Table 1. The conductivity of the deionized water that was used for the dialysis and sample preparation was 2 μS/cm after equilibration with atmospheric CO_2_.

**Table 1:** Molecular Weights of PDADMAC and PAAcDMAC Samples Used in This Study

commercial name	molecular weight (Da) reported by the manufacturer	mole fraction of DADMAC^c^	*f* ^d^
PDADMAC			
PDADMAC^a^	400,000–500,000	1.00	0.27
Merquat 100	150,000	1.00	0.30
PAAcDMAC			
Merquat 740	100,000	0.22	0.08
Merquat 550^b^	1,600,000	0.17	0.05
Merquat 2200^b^	1,600,000	0.16	0.06

a From Sigma-Aldrich, CAS no. 26062-79-3.

b Merquat 2200 and 550 are the same copolymer of the same molecular weight. Merquat 2200 is the dried, preservative free version, and this sample is differentiated from Merquat 550 by the PF label in the text.

c The mole fractions are obtained from ^1^H NMR (Figure S1 of the Supporting Information).

d Obtained by fitting osmotic pressure data to eq 3 with relative uncertainty in *f* of ±10%.

### 3.2 Purification

The samples were dialyzed by ultrafiltration. Deionized water was flushed through a 400 mL Amicon stirred cell (Millipore UFSC40001) under 30 psi of argon. Ultrafiltration membranes (Ultracel) with a molecular weight cutoff (MWCO) of 3 kDa were used. Dialysis continued until the conductivity of the cell outlet approached that of the deionized water. The conductivity of the dialysate was measured at room temperature using an Oakton COND 6+ Conductivity Meter. An additional advantage of ultrafiltration is that, unlike dialysis using dialysis tubing immersed in a water bath, it does not lead to replacement of some of the Na^+^ counterions of polyanions, such as NaPSS, with H^+^ ions caused by CO_2_ absorption from the atmosphere.^25^ After dialysis, the solutions were freeze-dried to remove water. The resulting powder was stored in a vacuum oven at 25 °C overnight to eliminate residual moisture, and the dried polymer was then used to prepare the solutions.

Figure 2 shows dialysate conductivity versus dialysis time. For all samples, the initial effluent exhibited a conductivity of order 1000 μS/cm. The feed polycation solutions were prepared by dissolving 1 to 2 g of each polymer sample in 300 mL of deionized water. Despite differences in polymer concentration, all samples had a pronounced decrease in dialysate conductivity by about 200 h, indicating removal of residual salts. Thereafter, the conductivity decay slowed and converged to similar steady levels for all samples, beyond which no further change was observed. The contribution from dissolved CO_2_, which perhaps reacts with water to form carbonic acid,^26^ sets the final baseline for the conductivity of the dialysate. Ions leached from the glass containers used to collect the dialysate may also contribute to this baseline.^8^

**Figure 2:**
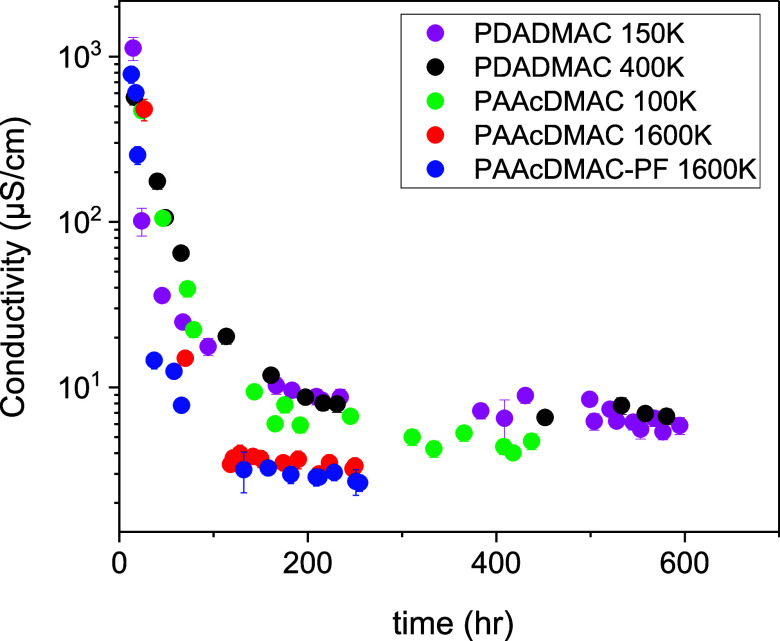
Time dependence of dialysate conductivity for PDADMAC and PAAcDMAC samples. Complete dialysis requires about 200 h for these samples. The uncertainty is smaller than the symbol size unless otherwise indicated.

### 3.3 Viscosity Measurements

Viscosity measurements were made using Cannon-Ubbelohde viscometers (size Nos. 50, 75, 150, and 200; Cannon Instrument Co.). The viscometers were held vertically in a constant-temperature water bath using a viscometer holder. The bath temperature was maintained at 25 °C. For each concentration, three replicate measurements were taken, and the flow times were averaged to determine the solution viscosity. Viscosity measurements for the high concentration samples were performed using a strain-controlled Rheometrics Fluids Spectrometer (RFS-III) rotational rheometer and a stress-controlled Kinexus Ultra rheometer (NETZSCH, Germany). For the RFS-III measurements, a concentric cylinder geometry was used, with inner and outer cylinder diameters of 16.5 mm and 17.0 mm, respectively, and an inner cylinder height of 13.0 mm. The inner cylinder is fully submerged in the solution for both calibration and all measurements. For the Kinexus Ultra measurements, a cone and plate geometry with a 40 mm diameter and 1° cone angle or a double-gap concentric cylinder geometry was used, as appropriate. The double-gap geometry consisted of a bob and a lower fixture. The bob had inner and outer diameters of 23 and 25 mm, respectively, and an internal wall height of 59.5 mm. The lower fixture had a cup diameter of 26.25 mm, an insert diameter of 21.9 mm, and an insert height of 58.5 mm. A solvent trap containing deionized water was used to minimize evaporation. All rheological measurements were carried out at 25 °C using the temperature control system of each rheometer.

### 3.4 Osmotic Pressure Measurement

Osmolality of the solutions was measured using a freezing point depression osmometer (OsmoTECH XT, Advanced Instruments). Because freezing point depression is a colligative property, the measured osmolality depends on the total number density of dissolved species, including unattached ions. In these solutions, this method effectively determines the sum of the number density of dissociated polymer counterions and the number density of salt ions.^27^

### 3.5 Dielectric Relaxation Spectroscopy

190 μL of the liquid sample was placed in a custom-built cylindrical stainless-steel cell with an electrode spacing of 1.63 mm. The gap between the electrodes was controlled by a sapphire window with electrode diameter of 10 mm. The sample was mounted in a Novocontrol Technologies BDS 1200 sample chamber (Montabaur, Germany). Dielectric measurements were conducted using a Novocontrol Alpha High-Resolution broadband dielectric/impedance spectrometer (Montabaur, Germany) with a 0.1 V excitation amplitude and no applied DC bias. Isothermal frequency sweeps were recorded over the range 10^7^ to 10^–1^ Hz. Conductivity is determined by averaging the in-phase conductivity over the range where it is nearly independent of frequency (see Supporting Information Figure S2).

### 3.6 Small Angle X-ray Scattering

SAXS measurements were carried out at the BL40B2 beamline of the SPring-8 synchrotron (Hyogo, Japan). The sample-to-detector distance was 2.2 m and the X-ray energy was 12.4 keV. Solutions were loaded into 2 mm glass capillaries and sealed with a glue gun to prevent evaporation. The capillaries were placed on a metal holder, and the temperature was maintained at 25 °C using a Peltier system. Scattering and transmission measurements were carried out simultaneously. Acquisition times were between 30 and 120 s. More details are provided in Table S1 of the Supporting Information.

### 3.7 ^1^H NMR

An 850 MHz Bruker Avance III NMR spectrometer equipped with a 5 mm microimaging probe was used to acquire the ^1^H NMR spectrum of PDADMAC and the PAAcDMAC copolymers dissolved in D_2_O. A relaxation delay of 5 s was applied during NMR measurements to ensure precise integration values.

## 4 Results and Discussion

### 4.1 Viscosity

Figure 3 shows the concentration dependence of specific viscosity for dialyzed and not dialyzed PDADMAC and PAAcDMAC aqueous solutions. In the dilute regime, the specific viscosity of as-received PDADMAC and PAAcDMAC scales linearly with concentration (η_sp_ ∼ *c*_p_). In the semidilute unentangled regime, the dialyzed and as-received PDADMAC homopolymers and copolymers follow the expected Rouse scaling, η_sp_ ∼ *c*_p_^1/2^.^1,5^

**Figure 3:**
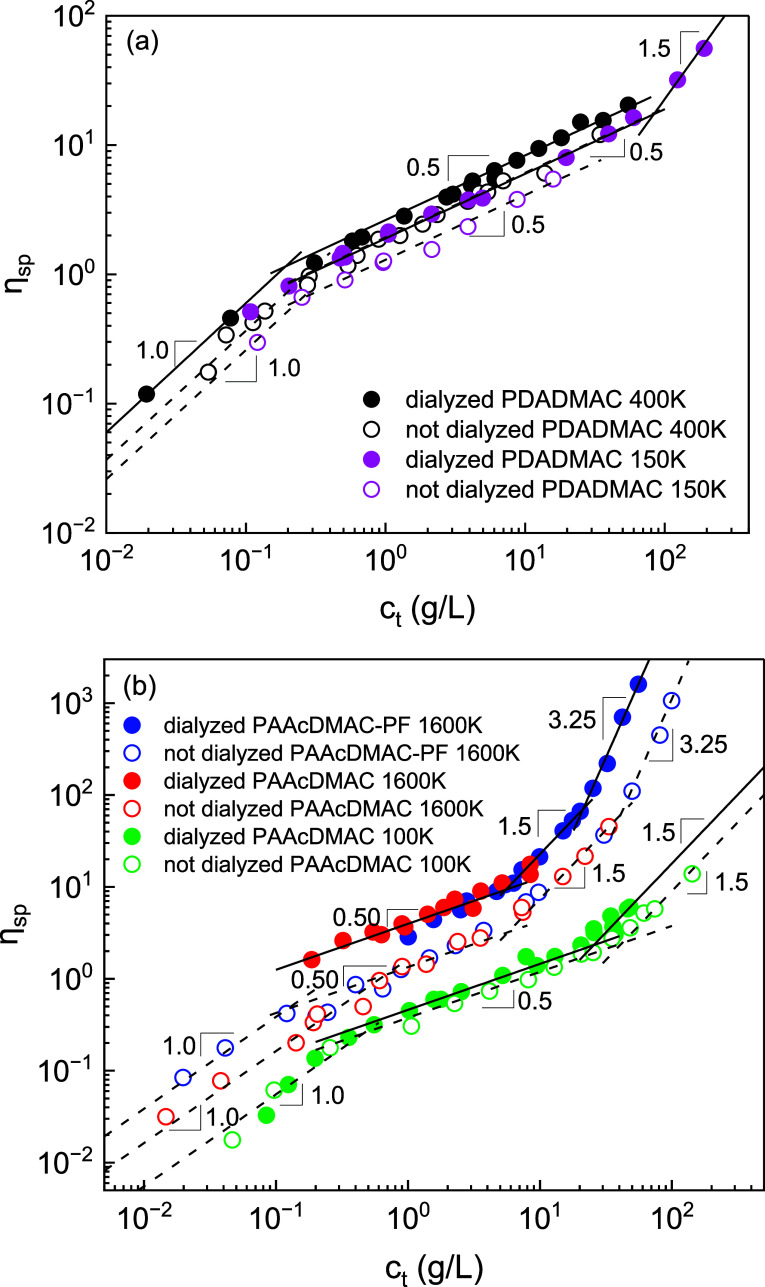
Concentration dependence of specific viscosity for dialyzed (filled symbols) and not dialyzed (open symbols) (a) PDADMAC and (b) PAAcDMAC aqueous solutions at 25 °C. Concentration, *c*_t_, is the mass of the weighed dry samples per liter. For both panels (a,b), the uncertainty is smaller than the symbol size.

As predicted by scaling theory, added salt lowers the specific viscosity, eq 2. In the high-salt limit of an unentangled semidilute polyelectrolyte, η_sp_ follows the scaling prediction of η_sp_ ∼ *c*_p_^5/4^ *c*_s_^–3/4^.^5,28^ In Figure 3, *c*_t_ is the total sample concentration in mass per volume. For dialyzed samples this corresponds to polymer only, while for the as-received samples it includes polymer plus residual salts. For the as-received samples, the polymer and residual salt contributions are initially unknown, so the concentrations are expressed in terms of *c*_t_, and the ratio *c*_s_/*c*_p_ is subsequently determined from osmotic pressure and conductivity measurements. For the as-received samples *c*_s_/*c*_p_ is constant; therefore, for the not dialyzed samples in Figure 3, the concentration dependence is expected to follow the same power-law scaling as the dialyzed samples, η_sp_ ∼ *c*_p_^1/2^, with a smaller prefactor.

Liberatore and co-workers^10^ have studied the 400–500 kDa PDADMAC and 1600 kDa PAAcDMAC-PF (Merquat 2200) samples without purification. The specific viscosity values they report for Merquat 2200 in deionized water are close to those measured in this work for not dialyzed samples, indicating that their “no added salt” data already reflect the presence of significant residual salts (Figure S3 of the Supporting Information). Residual salts reduce coil size, lowering the zero-shear viscosity in the dilute, semidilute unentangled, and entangled regimes, and shift the overlap concentration (*c*_p_*) to higher values.^29^ Therefore, since their polymer solutions prepared with deionized water contain residual ions, the shift in rheological properties at a given polymer concentration upon adding 0.1 M NaCl is smaller than the shift when starting from a truly salt-free dialyzed solution.^10^

### 4.2 Osmotic Pressure

In polyelectrolyte solutions, osmotic pressure is dominated by salt ions and counterions, eq 3. Consequently, the presence of residual salt increases the osmotic coefficient. The ideal osmotic pressure is calculated for a solution in which the counterions do not interact with each other or with the macroion. Therefore, φ can be used to assess the fraction of osmotically active counterions in dialyzed samples with no added salt. In the low salt limit, each dissociated counterion contributes *k*_B_ *T* to the osmotic pressure, given by π = *fk*_B_ *Tc*_p_, so φ can be approximated as φ ≃ *f* for semidilute solutions.^30^ For a solution containing both polyelectrolyte and salt, eq 3 can be rewritten for the osmotic coefficient in terms of the ratio of salt to monomer concentration, *c*_s_/*c*_p_, as^1^

5 $$\frac{\pi }{k_{B}Tc_{p}}=\varphi =\frac{f^{2}}{f+4c_{s}/c_{p}}+2c_{s}/c_{p}.$$

The value of *f* can be obtained from the osmotic coefficient of the dialyzed polyelectrolyte solutions with no added salt, and assuming that *f* is independent of the residual salt content, *c*_s_/*c*_p_ can then be calculated using eq 5 for the as-received samples. This approximation enables estimation of the residual salt content from the osmotic coefficient data. Since *f* might decrease with increasing added salt concentration, the calculated *c*_s_/*c*_p_ values may be slightly underestimated and should be considered as estimates.^31^

The osmotic coefficients of dialyzed and not dialyzed PDADMAC and PAAcDMAC aqueous solutions are plotted in Figure 4. The average osmotic coefficient for the dialyzed samples, indicated by the horizontal lines in Figure 4, corresponds to the *f* value for each sample. The fraction of free counterions is in the range of 0.27–0.30 for the homo-PDADMAC samples and in the range of 0.05–0.08 for the copolymers. The considerably lower *f* values for the copolymers, compared to homo-PDADMAC can be attributed to their low DADMAC content reported in Table 1, as determined by ^1^H NMR (Figure S1 of the Supporting Information).

**Figure 4:**
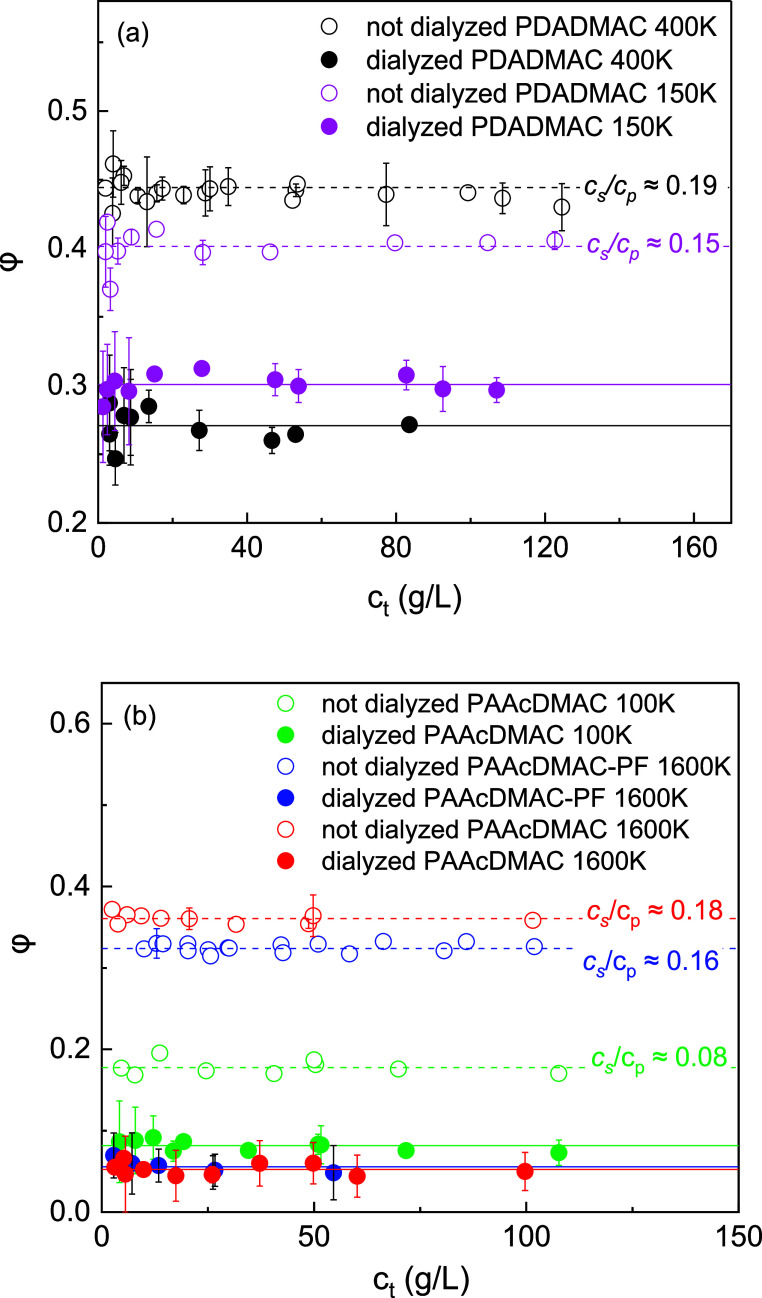
Osmotic coefficient of dialyzed (filled symbols) and not dialyzed (open symbols) (a) PDADMAC and (b) PAAcDMAC aqueous solutions at room temperature. Uncertainty is shown where it exceeds the symbol size.

The corresponding *c*_s_/*c*_p_ for each sample are shown in Figure 4 for the not dialyzed samples. For PAAcDMAC 100K, the value of *c*_s_/*c*_p_ is calculated to be nearly half that of PDADMAC samples and the higher molecular weight copolymers, which is in qualitative agreement with viscosity measurements, showing that PAAcDMAC 100K exhibits the smallest increase in specific viscosity after dialysis.

### 4.3 Conductivity

Comparison of the conductivity, σ, of the dialyzed and as-received PDADMAC and PAAcDMAC samples is presented in Figure 5. Conductivity versus frequency spectra for all samples, with σ extracted from the frequency-independent plateau region, are shown in Figure S2 of the Supporting Information. As expected, the not dialyzed samples show significantly higher conductivity than the dialyzed samples. The crossover from a low-*c*_p_ plateau, σ ∼ *c*_p_^0^, to the linear increase, σ ∼ *c*_p_^1^, provides an estimate of the residual salt concentration in the solvent, *c*_res,s_, for each sample.

**Figure 5:**
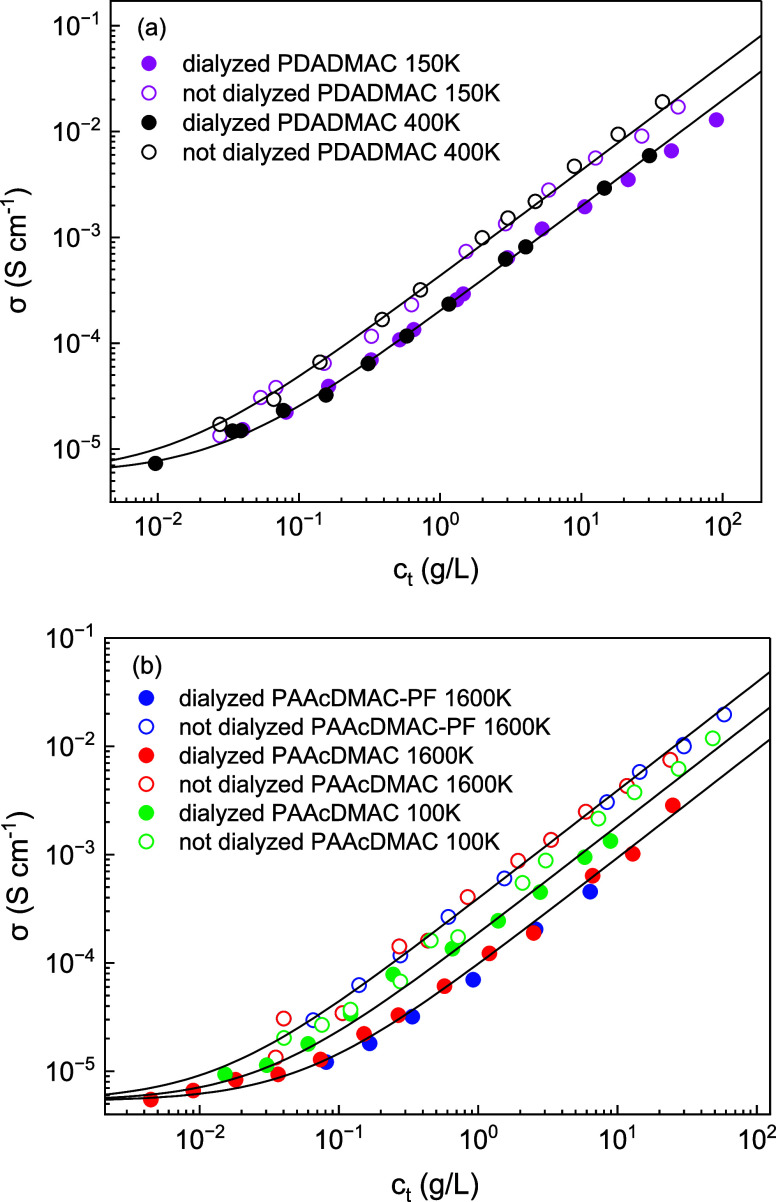
Ionic conductivity of dialyzed (filled symbols) and not dialyzed (open symbols) (a) PDADMAC and (b) PAAcDMAC aqueous solutions at room temperature. The solid curve is a fit to $\sigma =\sigma_{0}\left(1+\frac{c_{t}}{c_{res,s}}\right)$ , where σ_0_ is the solvent conductivity, and *c*_res,s_ is the residual salt content in the solvent. For both panels (a,b), the uncertainties are smaller than the symbol size.

The value of *c*_s_/*c*_p_ can also be estimated from conductivity data. The ionic conductivity of a polyelectrolyte solution depends on the number and mobility of all charged species present. In this study, the residual salt is assumed to be NaCl for PDADMAC and PAAcDMAC. The total conductivity is the sum of contributions from Na^+^, Cl^–^, the polyelectrolyte and its Cl^–^ counterions. The limiting molar conductances of Na^+^ (Λ_Na_^+^ = 50.1 S cm^2^ mol^–1^) and Cl^–^ (Λ_Cl_^–^ = 76.3 S cm^2^ mol^–1^) at 25 °C are used, giving a combined value of 126.4 S cm^2^ mol^–1^ for NaCl.^32^ With ionic interactions neglected at millimolar salt levels, this limiting value is used to estimate the residual salt concentration in the as-received samples. The differences in conductivity between dialyzed and not dialyzed solutions at the same polymer concentration give *c*_s_/*c*_p_ of 0.25 for PAAcDMAC 1600K and PAAcDMAC-PF 1600K, 0.07 for PAAcDMAC 100K, and 0.44 for both molecular weights of the PDADMAC samples. Table 2 compares *c*_s_/*c*_p_ from osmotic pressure and conductivity data for the as-received samples.

**Table 2:** Values of *c*_s_/*c*_p_ in the as-received Samples Estimated from Different Methods^a^

	*c*_s_/*c*_p_ estimated by	
sample	π	σ
PAAcDMAC-PF 1600K	0.16	0.25
PAAcDMAC 1600K	0.18	0.25
PAAcDMAC 100K	0.08	0.07
PDADMAC 400K	0.19	0.44
PDADMAC 150K	0.15	0.44

a The uncertainty in *c*_s_/*c*_p_ is 6 × 10^–3^ for the osmotic pressure method and 1 × 10^–4^ for the conductivity method.

Each method used to estimate *c*_s_/*c*_p_ has limits that can bias the result. For the osmotic pressure analysis, the assumption that *f* does not change with residual salt content can affect the estimated *c*_s_/*c*_p_, and this effect is more apparent for the homo-PDADMAC samples because their larger *f* values make the calculation more sensitive to the assumed *f*. For conductivity analysis, we used the NaCl limiting molar conductance at 25 °C, so finite-concentration corrections are ignored. Furthermore, discrepancies in *c*_s_/*c*_p_ estimates between osmotic pressure and conductivity methods may also arise from technique-specific artifacts, such as residual salt contamination introduced by contact with the stainless-steel surface of the DRS liquid cell.^33^

### 4.4 Correlation Length

Figure 4 shows that the copolymers have smaller *f* than the homo-PDADMAC samples. In salt-free semidilute polyelectrolyte solutions, a lower *f* increases the effective spacing between charges along the backbone, which weakens electrostatic repulsion between chains and leads to a larger structural correlation length, ξ, at a fixed concentration. The SAXS profiles for dialyzed PDADMAC and PAAcDMAC aqueous solutions are provided in Figure S4 of the Supporting Information. The extracted correlation lengths, ξ = 2π/*q*_max_, from the peak position (at wavevector *q*_max_) of the scattering profile at each concentration, together with the best-fit power laws are shown in Figure 6a.

**Figure 6:**
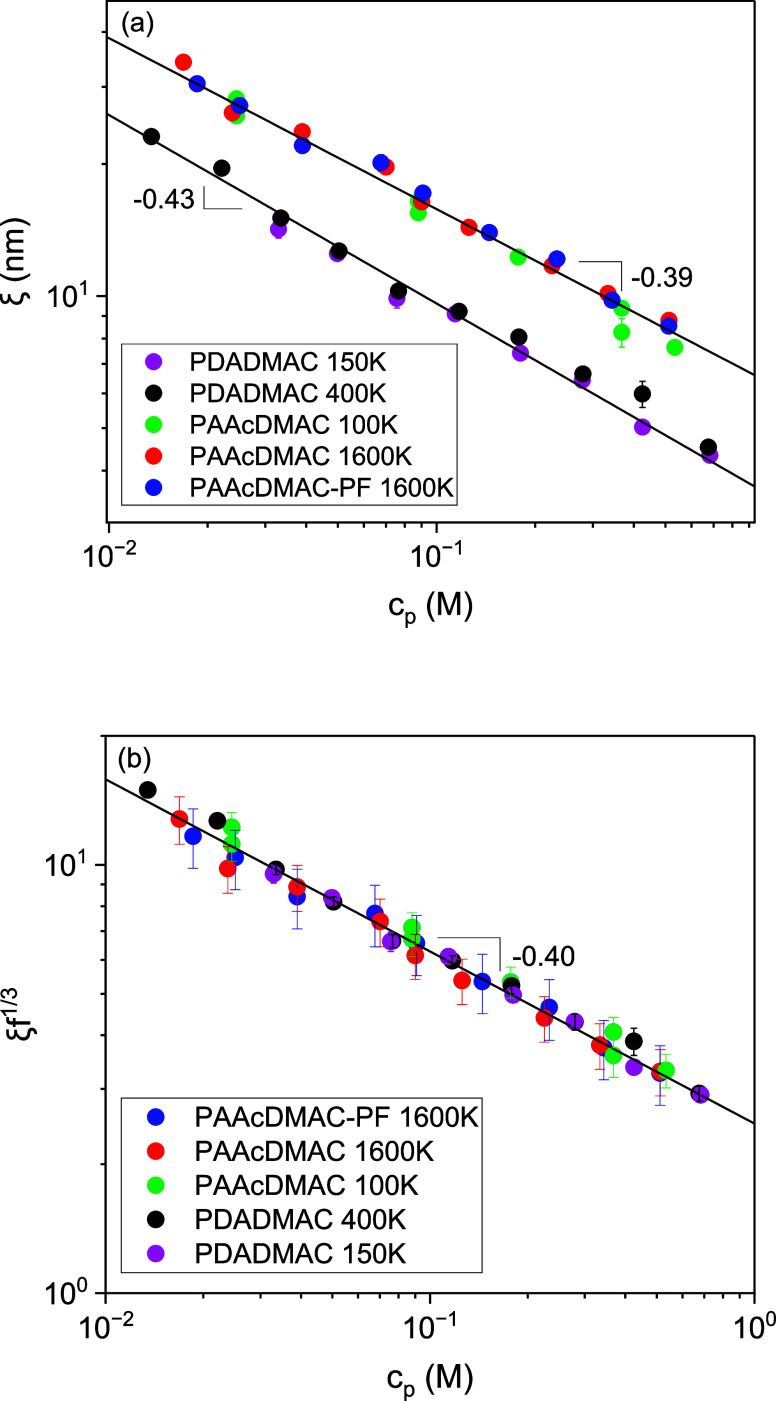
(a) Correlation length and (b) reduced correlation length (*f* ^1/3^ξ) versus *c*_p_ for dialyzed PDADMAC and PAAcDMAC aqueous solutions at 25 °C. The value of ξ at each concentration was obtained from SAXS profile peaks (Supporting Information, Figure S4).

According to scaling theory for salt-free semidilute polyelectrolytes, the correlation length scales as ξ ∼ *c*_p_^–1/2^, where the prefactor depends on the chain contraction factor, *B*. The −1/2 scaling is common, but not universal, and smaller exponents have been reported for several polyelectrolytes, including mucin bottlebrush polyelectrolytes, proteoglycan molecules from cartilage, polyionene solutions with certain counterions, star-branched polyelectrolytes, and dendrimer polyelectrolytes.^34^ These deviations have been associated with factors such as strongly hydrating side groups, counterion or polymer hydration, and compact or branched polyelectrolyte architectures.^34^

In the θ-solvent limit, *B* can be expressed as *B* ≈ *f* ^−2/3^(*b*/*l*_B_)^1/3^, where *l*_B_ is the Bjerrum length, and putting this into the low salt limit of eq 1 yields *f* ^1/3^ξ ∼ *c*_p_^–1/2^.^5^ Consequently, *f* ^1/3^ξ versus *c*_p_ collapses the data from samples with different free-ion fractions, *f*, listed in Table 1. Similarly, Nishida et al.^35^ found that in semidilute aqueous solutions of the sodium salt of partially sulfuric acid esterified poly­(vinyl alcohol) with no added salt, the correlation length scales as ξ ∼ *f* ^–1/3^ *c*_p_^–1/2^, in the regime without counterion condensation (*a* < 0.3), where *a* is the degree of esterification, and *f* ≈ *a*.^35^ The fit in Figure 6b for PDADMAC and PAAcDMAC samples, however, gives a weaker concentration dependence than the expected *c*_p_^–1/2^ scaling.

The weaker concentration dependence of ξ than that predicted by scaling theory for both the homo-PDADMAC and the copolymers in Figure 6a might be attributed to chain branching in PDADMAC. Commercial PDADMAC may contain branched chains due to side reactions during cyclopolymerization of diallyldimethylammonium chloride, where pendant vinyl groups may react and form branch points.^36^ Branching and cross-linking in PDADMAC have been reported to depend on the reaction temperature and time and were attributed to polymerization of residual monomer and reactions involving unreacted double bonds.^37^ Wandrey and Freitag demonstrated that specialized synthesis routes are required to obtain strictly linear, narrowly distributed PDADMAC with reduced branching, in contrast to the more heterogeneous commercial PDADMAC.^36^ Xia et al.^38^ also reported that as molecular weight increased, fractionated PDADMAC shows a decreasing shape factor, *p* = *R*_g_/*R*_h_, where *R*_g_ is the radius of gyration and *R*_h_ is the hydrodynamic radius. They showed downward curvature in the molecular weight dependence of both intrinsic viscosity and radius of gyration at high molecular weight, and attributed both trends to branching in the high molecular weight tail of the PDADMAC.^38^ The *R*_g_ measurements of PDADMAC in aqueous 0.3 M NaNO_3_ were reported to exhibit a scaling exponent of 0.454 for *R*_g_ versus molecular weight. This weaker dependence of *R*_g_ on molecular weight compared with the exponent expected for a linear flexible chain under screened electrostatic conditions is consistent with branching in PDADMAC.^39^ Such branching and cross-linking can reduce the effective chain extension and alter interchain correlations, consistent with the observed deviation from the ideal −0.5 exponent in Figure 6. Similar deviations from the linear polyelectrolyte concentration scaling have been reported for aqueous star-branched polyelectrolyte solutions with no added salt.^40–42^ Boué et al. found that the first scattering maximum scales as q* ∼ *c*_p_^1/3^ for star-branched NaPSS, rather than the q* ∼ *c*_p_^1/2^ scaling associated with the polyelectrolyte peak of semidilute linear NaPSS, indicating that branched architecture can change the concentration scaling of the scattering maximum.^40^

## 5 Conclusion

Removal of residual salts is necessary for accurate assessment of polyelectrolyte solution properties, yet many studies do not dialyze commercial polyelectrolyte samples, leading to overlooked salt effects and underestimation of their impact on key solution properties. Dialysis of commercial polyelectrolytes yields reproducible rheological and osmotic measurements and conductivity data that reflect intrinsic polymer behavior rather than uncontrolled ionic impurities. This work shows that purification substantially alters solution viscosity, the osmotic coefficient, and ionic conductivity in commercial homo-PDADMAC and its copolymer with acrylamide. Being a readily available polycation, PDADMAC has often been used in polyelectrolyte complexes and coacervates by blending with a polyanion.^43–46^ The possible branching of PDADMAC may affect the interpretation of association, hydration, swelling, structure, rheology, and the unexpected asymmetry of coacervates made using PDADMAC.^43,44^

## Supplementary Material





## Notes

Excel data sheet with labeled columns, containing the data associated with the figures in the main text and the Supporting Information (XLSX)
^1^H NMR spectra of PAAcDMAC 100K, PAAcDMAC 1600K, PAAcDMAC-PF 1600K, PDADMAC 150K, and PDADMAC 400K in D_2_O. Conductivity versus frequency spectra for aqueous solutions of dialyzed and not dialyzed PDADMAC 150K, dialyzed and not dialyzed PDADMAC 400K, dialyzed and not dialyzed PAAcDMAC 100K, dialyzed and not dialyzed PAAcDMAC 1600K, dialyzed and not dialyzed PAAcDMAC-PF 1600K at different concentrations, *c*_t_. Comparison of the concentration dependence of specific viscosity for aqueous solutions of not dialyzed PAAcDMAC-PF 1600K from this work and reported in the literature.^10^ The SAXS profiles for dialyzed PDADMAC 150K, PDADMAC 400K, PAAcDMAC 100K, PAAcDMAC 1600K, and PAAcDMAC-PF 1600K at different concentrations. Transmission, capillary thickness, and acquisition time for dialyzed aqueous PDADMAC and PAAcDMAC solutions used in the SAXS measurements (PDF)
