# Supplementary material for: Effect of Dialysis on the Osmotic Pressure, Conductivity, and Rheology of Aqueous Polyelectrolyte Solutions
Source: ACS Appl Polym Mater. 2026 Jun 15;8(12):9288–96. doi: 10.1021/acsapm.6c00957 (PMC13316864; doi:10.1021/acsapm.6c00957)
Supplement: Supplementary file 2 [file ap6c00957_si_002.pdf]

Supporting Information:

Effect of Dialysis on the Osmotic Pressure, Conductivity, and Rheology of Aqueous Polyelectrolyte Solutions

Bahar Baniasadi [1], Arva Tejas Desai [2], Zitan Huang [3], Victoria Devine-Ducharme [4], Carlos G. Lopez [3], Ralph H. Colby [1,3*]*

1. Department of Chemical Engineering, The Pennsylvania State University, University Park, PA 16802.

2. Department of Mechanical Engineering, The Pennsylvania State University, University Park, PA 16802.

3. Department of Materials Science and Engineering, The Pennsylvania State University, University Park, PA 16802.

4. Department of Biomedical Engineering, The Pennsylvania State University, University Park, PA 16802.

* Email: cvg5719@psu.edu, *Carlos G. Lopez*

* Email: rhc@plmsc.psu.edu, *Ralph H. Colby*

^{1}H NMR analysis of PAAcDMAC

The molar ratio of PDADMAC to acrylamide in the copolymers was determined by quantitative ^{1}H NMR. Figure S1 shows the assigned ^{1}H NMR spectra with the integration regions. Peak assignments followed references [1] and [2].

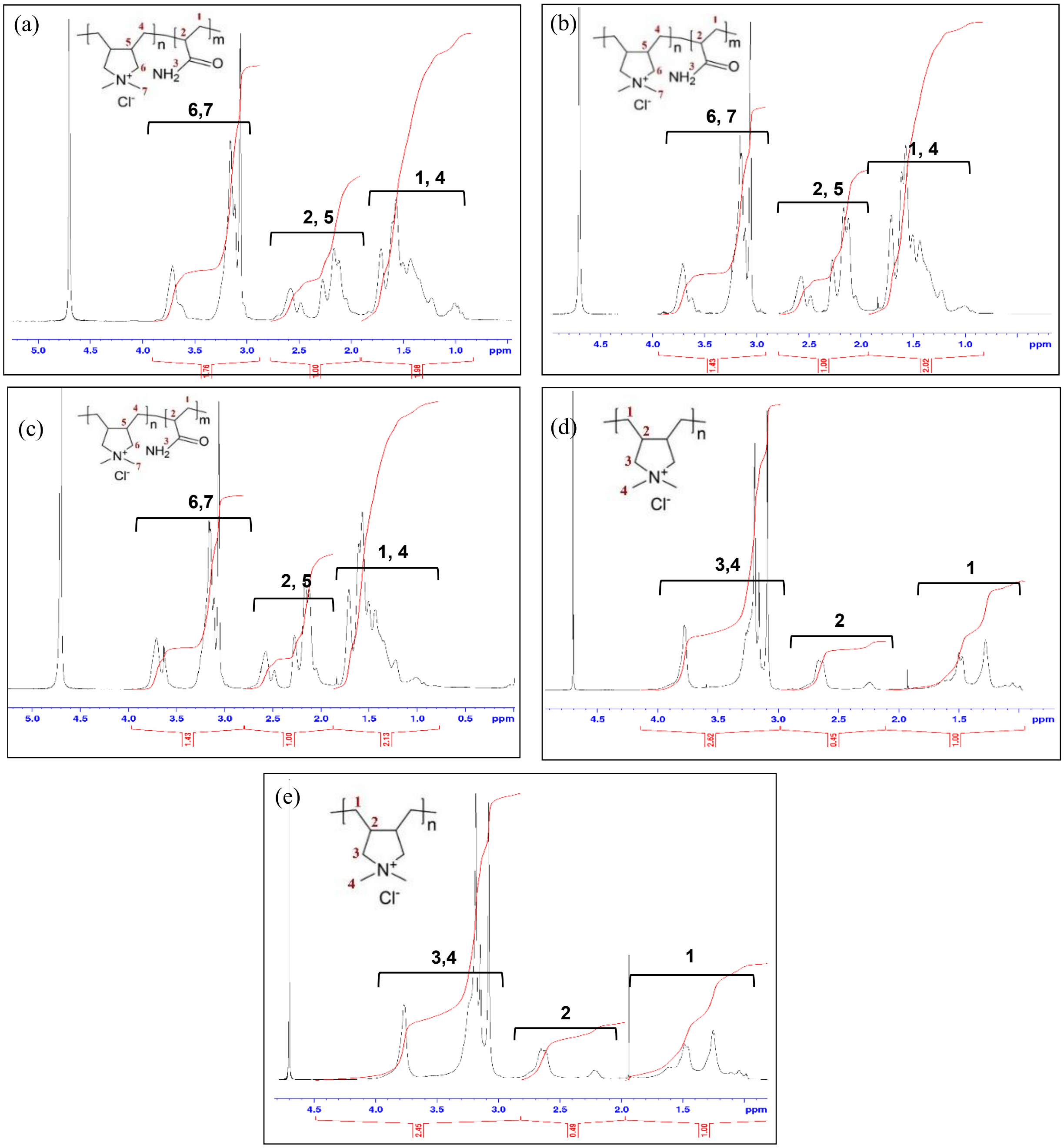

Figure S1. ^{1}H NMR spectra of (a) PAAcDMAC 100K, (b) PAAcDMAC 1600K, (c) PAAcDMAC-PF 1600K, (d) PDADMAC 150K, and (e) PDADMAC 400K in D_2O. For copolymers using the equations, $\frac{\int 6,7}{\int 2,5} = \frac{10n}{2n+m}$ and $\frac{\int 1,4}{\int 6,7} = \frac{2m+4n}{10n}$ gives a molar ratio of acrylamide to DADMAC of 3.64, 5.06, and 5.21 for the 100K PAAcDMAC, 1600K PAAcDMAC, and 1600K PAAcDMAC-PF, respectively.

Dielectric Relaxation Spectroscopy (DRS):

Conductivity spectra from DRS for dialyzed (filled symbols on left) and not dialyzed (open symbols on right) PDADMAC and PAAcDMAC aqueous solutions over 10^7 to 10^{-1} Hz at room temperature are shown in Figure S2. At lower frequencies, electrode polarization leads to an apparent decrease in the measured conductivity with $\sigma \sim \mathrm{frequency}$. This effect is well known and arises from the accumulation of charges at the electrode interface, which lowers the effective electric field experienced by the sample. The plateau value at intermediate frequencies was taken as the DC conductivity (σ) and reported in Figure 5 of the main text.

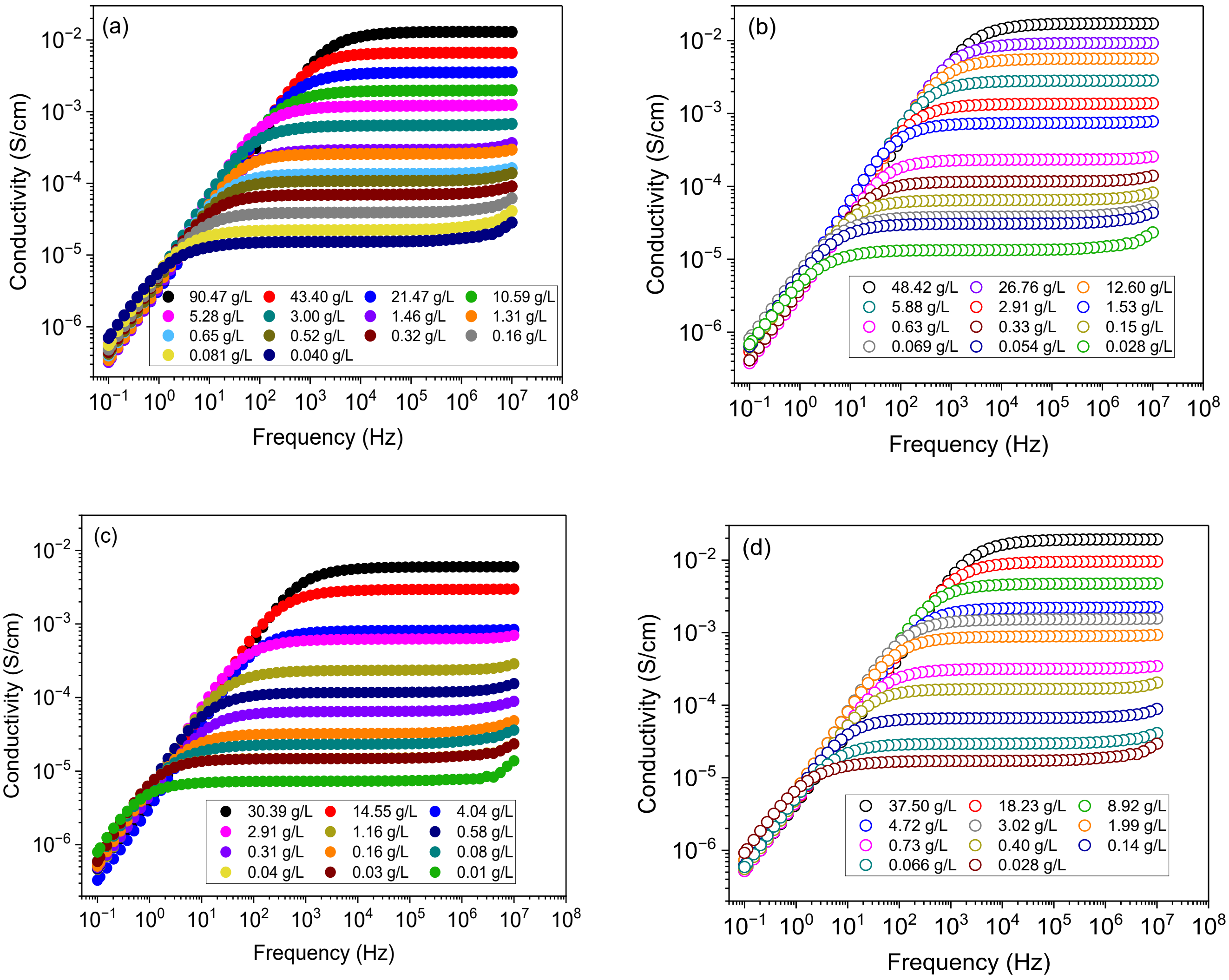

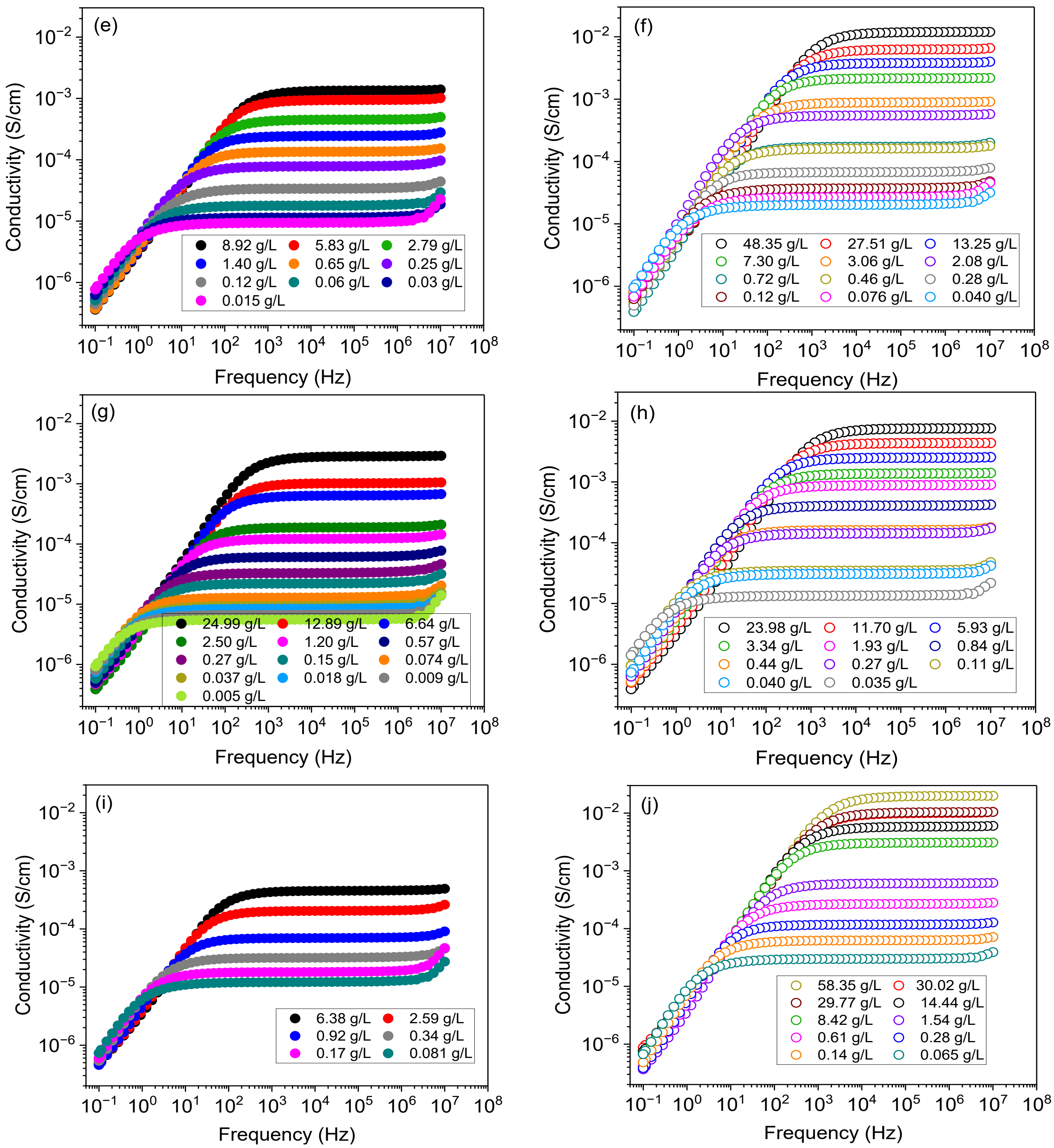

Figure S2. Conductivity versus frequency spectra for aqueous solutions of (a) dialyzed PDADMAC 150K, (b) not dialyzed PDADMAC 150K, (c) dialyzed PDADMAC 400K, (d) not dialyzed PDADMAC 400K, (e) dialyzed PAAcDMAC 100K, (f) not dialyzed PAAcDMAC 100K, (g) dialyzed PAAcDMAC 1600K, (h) not dialyzed PAAcDMAC 1600K, (i) dialyzed PAAcDMAC-PF 1600K, (j) not dialyzed PAAcDMAC-PF 1600K at different concentrations, c_t (listed in each legend)

Comparison of as-received PAAcDMAC-PF 1600K Viscosity Data with Literature

Figure S3 compares the specific viscosity of not dialyzed PAAcDMAC-PF 1600K in deionized water measured in this work with literature data reported by Liberatore and co-workers [3]. The agreement between the two data sets supports that the literature "no added salt" data correspond to the as-received, residual salt containing polyelectrolyte.

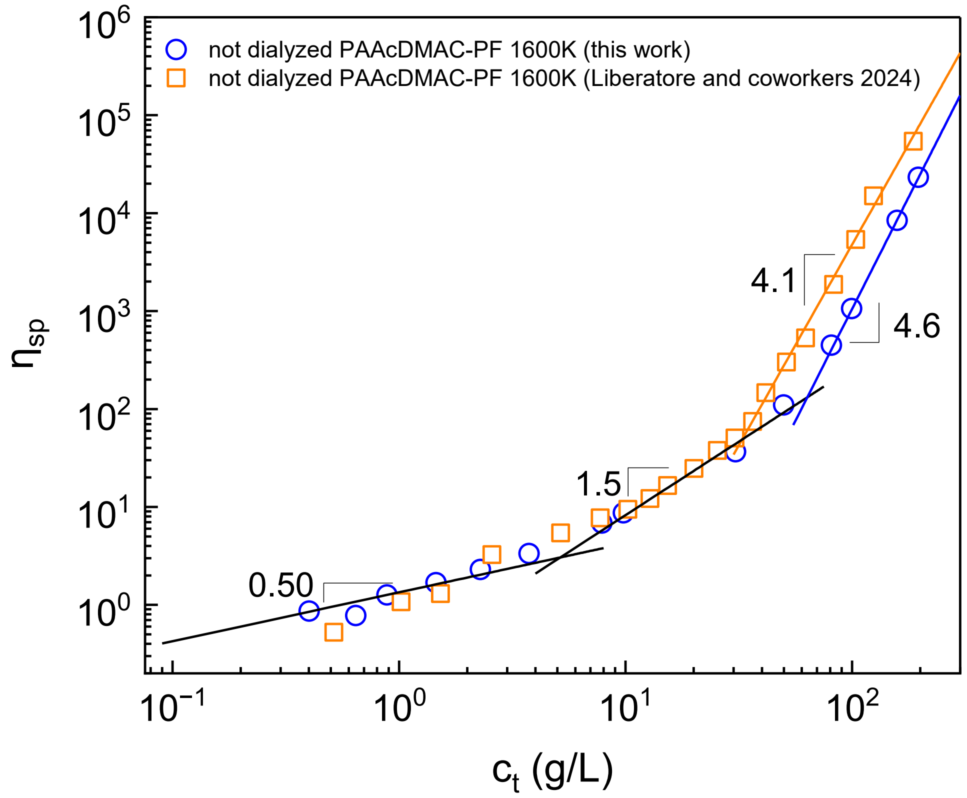

Figure S3. Comparison of the concentration dependence of specific viscosity for aqueous solutions of not dialyzed PAAcDMAC-PF 1600K from this work and reported by Liberatore and co-workers [3] at 25 °C. Concentrations reported in wt% in the literature were converted to g/L assuming a solution density of 1.02 g/mL, based on the density reported by Sigma-Aldrich for PAAcDMAC solution, 10 wt% in H_2O, at 25 °C [4].

Small-Angle X-Ray Scattering

Small-angle X-ray scattering profiles for aqueous solutions of dialyzed PDADMAC and PAAcDMAC samples after subtraction of the capillary signal are shown in Figure S4. The data were reduced using the thickness of the capillary tubes (t), the transmission (T) and the count rate for the sample and the empty capillary (I):

$$I(q) = \frac{I_{sample}(q)}{t_{sample}T_{sample}} - \frac{I_{empty\ capillary}(q)}{t_{sample}T_{empty\ capillary}}$$

where q is the magnitude of the scattering wavevector and $I(q)$ is the background subtracted scattering intensity. The subscripts, “sample” and “empty capillary” are for sample-filled and empty capillaries, respectively, and apply to all quantities in the equation. The values of the thickness of the capillary tubes, acquisition time, and transmission for each sample are provided in Table S1.

The position of the peak in the scattering profile gives $q_{max} = 2\pi/\xi$ which is used to determine the correlation length, ξ, plotted in Figure 6(a) of the main text.

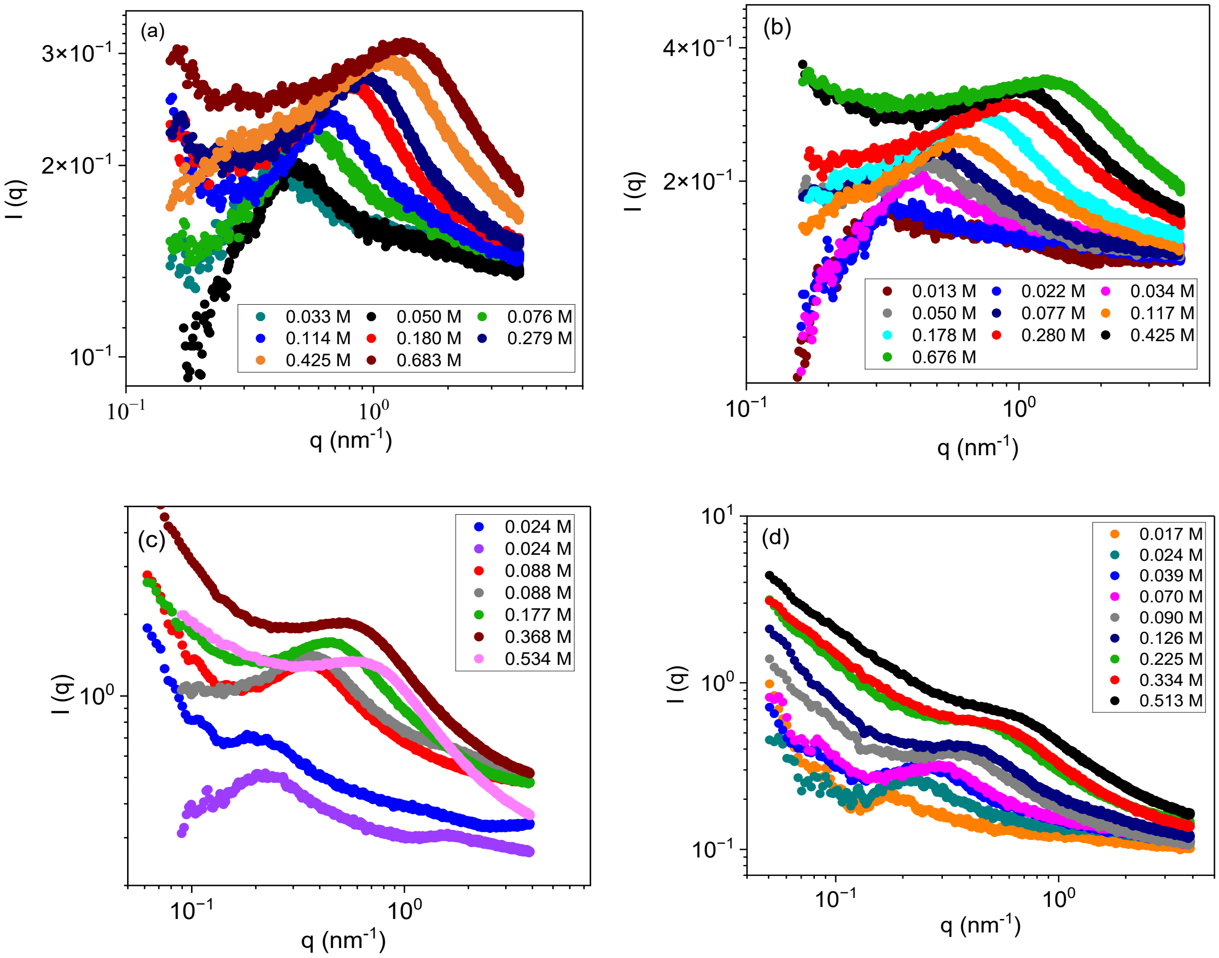

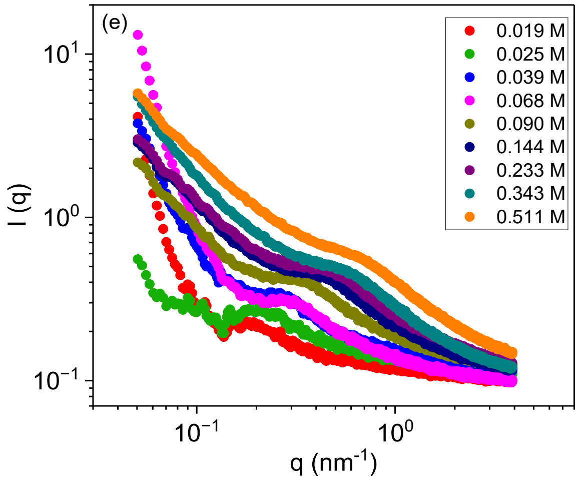

Figure S4. The SAXS profiles for dialyzed (a) PDADMAC 150K, (b) PDADMAC 400K, (c) PAAcDMAC 100K, (d) PAAcDMAC 1600K, and (e) PAAcDMAC-PF 1600K at different concentrations, c_p (listed in each legend). The far stronger peaks seen for homopolymer PDADMAC in Figure S4(a) and S4(b) stem from more Cl^- counterions (which dominate contrast) than the copolymers PAAcDMAC in Figure S4(c), S4(d), and S4(e).

Table S1. Transmission, capillary thickness, and acquisition time for dialyzed aqueous PDADMAC and PAAcDMAC solutions used in the SAXS measurements.

PDADMAC 150K

c_p (M)	transmission	capillary thickness (mm)	acquisition time (s)
0.033	0.578	1.85	60
0.050	0.591	1.75	60
0.076	0.594	1.75	60
0.114	0.615	1.65	60
0.180	0.591	1.7	60
0.279	0.583	1.7	60
0.425	0.554	1.8	60
0.683	0.556	1.7	60

PAAcDMAC 1600K

c_p (M)	transmission	capillary thickness (mm)	acquisition time (s)
0.017	0.632	1.65	30
0.024	0.631	1.65	30
0.039	0.617	1.65	30
0.070	0.633	1.65	30
0.090	0.635	1.65	30
0.126	0.619	1.65	30
0.225	0.577	1.65	30
0.334	0.61	1.65	30
0.513	0.579	1.65	30

PAAcDMAC 100K

c_p (M)	transmission	capillary thickness (mm)	acquisition time (s)
0.024	0.414	1.5	120
0.088	0.525	2.0	60
0.177	0.533	1.85	60
0.368	0.436	1.8	120
0.534	0.362	1.8	120

PDADMAC 400K

c_p (M)	transmission	capillary thickness (mm)	acquisition time (s)
0.013	0.629	1.65	60
0.022	0.623	1.65	60
0.034	0.597	1.6	60
0.050	0.635	1.5	60
0.077	0.611	1.6	60
0.117	0.606	1.65	60
0.178	0.559	1.8	60
0.280	0.583	1.7	60
0.425	0.575	1.7	60
0.676	0.602	1.5	60

PAAcDMAC-PF 1600K

c_p (M)	transmission	capillary thickness (mm)	acquisition time (s)
0.019	0.634	1.65	30
0.025	0.614	1.65	30
0.039	0.612	1.65	30
0.068	0.635	1.65	30
0.090	0.59	1.65	30
0.144	0.623	1.65	30
0.233	0.617	1.65	30
0.343	0.64	1.65	30
0.511	0.612	1.65	30

REFERENCES

[1] Abdollahi, M.; Ziaee, F.; Alamdari, P.; Koolivand, H. A comprehensive study on the kinetics of aqueous free-radical homo- and copolymerization of acrylamide and diallyldimethylammonium chloride by online ^{1}H-NMR spectroscopy. *J. Polym. Res.* 2013, 20, 239. DOI: 10.1007/s10965-013-0239-9

[2] Chee, C. H.; Benharush, R.; Knight, L. R.; Laaser, J. E. Segregative phase separation of strong polyelectrolyte complexes at high salt and high polymer concentrations. *Soft Matter* 2024, 20 (42), 8505–8514. DOI: 10.1039/D4SM00994K

[3] Helsper, S.; Singlar, N.; Garcia, A. G.; Liberatore, M. W. Viscosity scaling and entangled solution rheology in aqueous and salt solutions of polyelectrolytes containing diallyl dimethylammonium groups. *Rheol. Acta* 2024, 63 (2), 135–144. DOI: 10.1007/s00397-023-01428-6

[4] Sigma-Aldrich. Poly(acrylamide-co-diallyldimethylammonium chloride) Solution, 10 wt % in H2O; Product No. 409081; CAS No. 26590-05-6. *MilliporeSigma*.
